# Dietary supplement of *Acanthopanax senticosus* decoction formula improves immune response via intestine flora of rabbits

**DOI:** 10.3389/fmicb.2025.1508280

**Published:** 2025-03-04

**Authors:** Jing Nie, Qin Liu, Shihui Huang, Jiafu Wang, Xi Niu, Xueqin Ran

**Affiliations:** ^1^College of Animal Science, Guizhou University, Guiyang, Guizhou, China; ^2^Institute of Agro-Bioengineering, Guizhou University, Guiyang, Guizhou, China

**Keywords:** traditional medicinal herbs, immunity, rabbit, intestinal flora, inflammation

## Abstract

Young rabbits are sensitive to surrounding changes and conditioned pathogens in intestine which might result in slow inflammation and diarrhea after microbial invasion. Traditional medicine herbs could provide efficacious treatment on slow infection and inflammation. The present research designed an *Acanthopanax senticosus* (ACS) formula consisted of five types of Chinese herbs including *Acanthopanax senticosus* (Rupr & Maxim) Harms (Ciwujia in Chinese), *Astragalus membranceus* (Fisch) Bge (Huangqi in Chinese), *Indigo naturalis* (Qingdai in Chinese), *Houttuynia cordata* Thunb (Yuxingcao in Chinese), and *Glycyrrhizae radix* et Rhizoma (Gancao in Chinese). The effects of ACS decoction supplement were investigated via determination of cytokines and growth performances of young rabbits, and the flora in intestinal digesta from six fragments were further explored using 16S rRNA gene sequencing technology. Compared to the control group, rabbits supplied with different doses of ACS decoction possessed lower diarrhea and death rates, together with the IL-10 concentration, while the declined IL-1β and IL-12 levels and inflammatory factor gene expressions in intestinal tissues. Additionally, ACS addition changed the diversity of flora in each segments of intestine. Functional prediction on abundances of genera enriched to seven KEGG immunity pathways. Moreover, strong correlations were determined between the abundance of bacteria with interleukins contents, and the predictive immune signaling abundances, respectively. Especially, ACS exhibited anti-inflammation effects via decreasing the abundances of *Bacteroides*, *Clostridia_vadinBB60_group*, *NK4A214_group*, and *dgA_11_gut_group* in intestine of young rabbits. In conclusion, dietary supplement with ACS exerted diarrhea-reducing effects, and improved immunity homeostasis by modulating intestinal flora diversity in young rabbits.

## 1 Introduction

There are trillions of microorganisms in gastrointestinal tract, in which reside many immune cells and cytokines molecules involved in innate and adaptive immune systems to defend against bacterial invasion and maintain the intestinal health. The innate immune system is the first line of defense and consists of physical intestine barrier, chemical and cellular defenses. The adaptive immune response is the second gut defense and is pathogen-specific. Innate immunity occurs immediately while adaptive immunity develops upon pathogen exposure to produce antibody, and is long-lasting (Stambas et al., 2020). Both of them play synergistically effects in intestine defense processes against various harmful external agents and pathogenic invasion from the broken gut epithelial barrier (Zheng et al., 2020). It is clear that the prominent receptors of innate immunity are NOD-like receptors (NLRs) and Toll-like receptors (TLRs) in human gut. TLRs commonly combine with extracellular pathogens substances in gut, and most NLR members recognize the intracellular dangerous debris from pathogenic microbes (Zheng et al., 2020). Once these receptors of immune cells are activated, the intracellular signaling cascades involved in the inflammatory and other immune responses are rapidly induced, such as NOD-like receptor signaling, and Toll and lmd signaling pathway. Inflammasome fulfills a central role in the inflammatory process of innate immunity, which is a supramolecular complex in the cytosol. A canonical inflammasome comprises three parts (Fu et al., 2024): sensor, adaptor and effector proteins. The sensor protein, such as NLRP3, is triggered by intracellular danger signals of PAMPs (pathogen-associated molecular patterns) resulting in its oligomerization. Most inflammasome sensors are members of nucleotide-binding oligomerization domain-like receptor (NLR) family (Fu et al., 2024). And the adaptor protein, apoptosis-associated speck-like protein, is recruited by the sensor, and bridges the effector caspase-1. Caspase-1 then cleaved the proinflammatory cytokines to their matured forms of IL-1β and IL-18 (Fu et al., 2024).

Beside innate immunity, some stimuli from microbes in intestinal lumen can be recognized by adaptive immune system. FcγR is the core receptor in adaptive immune responses, which binds with extracellular pathogenic particles, activating phagosomes along with FcγR-mediated phagocytosis signaling. The engulfed materials in phagosomes are digested under the help of enzymes from the fused lysosomes. Then, the antigen-presenting cells, including dendritic cells (DCs) and phagocytes, are activated to present antigens via antigen processing and presentation pathway (especially by MHC-I and MHC-II molecules) and to produce IL-12 (Trinchieri, 2003). IL-12 participates in the immune cell mediated adaptive immunity via stimulating the differentiation of Th1 cells from naive CD4^+^ T cells. The primary effector of gut inflammatory response is CD4^+^ T cells. Th1 cells then induce strong cytotoxicity effects of macrophages and cytotoxic T cells to phagocytize those IgG-coated targets (Bournazos and Ravetch, 2015). In addition, the naive CD4^+^ T cells also differentiate into regulatory T (Treg) and Th17 cells. The differentiation of Th17 cells mainly dependent on IL-1β and aggravates inflammation by production of IL-17. Treg cells secret IL-10 and function as an inhibitor to Th1 and Th17 cells activities, decreasing the secretion of IL-1β and IL-17, thereby limiting inflammation and maintaining the immune homeostasis (Rasquinha et al., 2021).

Unavoidably, some conditional pathogenic bacteria residing in intestine might invade intestine especially those evading innate immune system by using a variety of strategies. After invasion, bacteria release macromolecules to further target components of NF-κB signaling pathway of intestinal epithelial cells, resulting in intestinal inflammation and diarrhea (Bruno et al., 2009). Thus, inhibiting inflammation and protecting intestinal epithelial integrity is considered to be an efficacious treatment for preventing pathogens invasion especially in young rabbits, which is more sensitive to seasonal changes due to their specific physiological characteristics, such as few sweat glands, thick coats of fur, high metabolic and growth rates (Ye et al., 2023a). The conditioned infections mostly encountered in intensive breeding farms caused by opportunistic organisms that act singly or jointly including bacteria, viruses, protozoa and mycetes. Furthermore, chronic, progressive and persistent infections might present in rabbits, which are slow, latent, tolerated, hidden infections or virus-associated tumors (Galassi et al., 1986). In commercial rabbits, diarrhea is an important factor influencing profitable production. During the late 2001 and early Spring of 2002 in Mexico, a sharp outbreaks of diarrhea characterized by a high morbidity and mortality occurred in different commercial rabbit farms. The most affected rabbits are aged 5–7 weeks with death rates varied from 16.7 to 35.2% (Rodríguez-De Lara et al., 2008). Similarly, the colibacillosis of rabbits caused by conditional pathogen *E. coli* occupy 42.8% from 2003 to 2011 in Guiyang, China (Wang et al., 2012). The traditional Chinese medicines can provide a strategy to deal with those disease caused by conditional pathogen via their anti-inflammation and immunity-modulation capability. Some herbal medicines are characterized by properties of against inflammation, oxidative stress, viral infection, and cancer (National Pharmacopoeia Committee, 2015), such as *Acanthopanax senticosus* (Rupr & Maxim.) Harms (Ciwujia in Chinese), *Astragalus membranceus* (Fisch.) Bge (Huangqi in Chinese), *Indigo naturalis* (Qingdai in Chinese), *Houttuynia cordata* Thunb (Yuxingcao in Chinese), and *Glycyrrhizae radix* et Rhizoma (Gancao in Chinese). And those herbs are widely used in Chinese medicine and are efficacious in treating various inflammatory diseases including ulcerative colitis and conditioned chronic inflammation caused by pathogens (Lau et al., 2019; Li et al., 2020; Liu X. et al., 2021; Kakdiya et al., 2024; Shu et al., 2024). However, interrelationship of these combined herbs with the intestinal flora remained largely unclear. This study designed an ACS decoction, which was combination of Ciwujia, Huangqi, Qingdai, Yuxingcao, and Gancao at different dosages, and investigated the effects of dietary ACS supplementation on the growth performance, intestinal immunity and intestinal flora structure in young rabbits.

## 2 Materials and methods

### 2.1 Experimental groups and management

A total 60 New Zealand white rabbits (weaned), with initial average body weight of 611.03 ± 71.05 g, 45-day-old, were randomly allocated to four groups with 3 replications for 5 rabbits per replication. The treatments were consisted of four groups (*n* = 15): control (NC) and low (DL), middle (DM) and high (DH) dosage groups supplemented with 4.3, 8.4, or 12.6 g/Kg of ACS (provided by TaiSheng Chinese Medicine Market in Guiyang, China). The ACS formula was designed based on “Jun-Chen-Zuo-Shi” rules (National Pharmacopoeia Committee, 2020), which included *Acanthopanax senticosus* (Rupr. & Maxim.) Harms (Ciwujia in Chinese), *Astragalus membranceus* (Fisch.) Bge (Huangqi in Chinese), *Indigo naturalis* (Qingdai in Chinese), *Houttuynia cordata* Thunb (Yuxingcao in Chinese), and *Glycyrrhizae radix* et Rhizoma (Gancao in Chinese) (Supplementary Table S1). Ingredients and compositions of the basal diet were shown in Supplementary Table S2. All rabbits were raised in vertical cages (60 cm × 40 cm × 40 cm) individually and were provided *ad libitum* access to fresh water and pellet feed. Temperature and lighting were maintained according to commercial conditions (20–23°C, 60–70% relative humidity, 12 light/12 dark). At the end of experiments (35 days), the rabbits were sacrificed by cervical dislocation, and the abdomen was immediately opened to take off the entire small intestine. The intestinal contents of duodenum, jejunum, ileun, cecum, colon, and rectum were collected and stored at −80°C firstly for 16S rRNA gene sequencing, and then segments (about 3 cm each) at the middle location were quickly isolated, and washed those intestinal segments gently by cold saline solution before stored at −80°C for further experiment. Furthermore, the digesta in each intestinal fragments were quickly collected as described in previous work (Chen et al., 2022), and stored at −80°C for microbiological analysis.

### 2.2 Growth performance and diarrhea

After 10 days of adaptive feeding, animals were feed ACS as mentioned above, the weight, feed intake, numbers of death and diarrhea of young rabbits were recorded. The numbers of diarrhea rabbits were collected based on the symptoms of soft or watery feces. The body weights of rabbits were measured after 12 h fasting on the morning at day 1 and day 35. The values of average daily body weight gain (ADG), the feed conversion ratio (FCR), the rate of mortality and diarrhea were then calculated. ADG = (Final body weight – Initial body weight)/Days. Diarrhea rate = [(numbers of diarrhea rabbits × diarrhea days)/(total numbers of rabbits × total days)] × 100%. FCR = Feed eaten (g)/Animal weight gain (g).

### 2.3 Blood cell counting and immune organ index detection

The blood samples were collected from the first to fifth week in the morning, and detected the numbers of blood cells as soon as possible. The rabbits were slaughtered at day 35, and the organs of spleen, thymus, sacculus rotundus and vermiform appendix were separated and weighed. The organ index was calculated as a ratio of the organ weight (g) to the body weight (kg).

### 2.4 Cytokines test in intestinal tissues

The concentrations of interleukin-10 (IL-10), interleukin-12 (IL-12), and interleukin-1β (IL-1β) in the intestinal tissues were determined by enzyme-linked immunosorbent assay (ELISA) kits from Jiangsu JingMei Biotechnology Co., Ltd. (JiangSu, China).

### 2.5 16S rRNA amplicon sequencing and analysis

The genomic DNA was extracted from the digesta in each intestinal fragment, and conducted quality inspection. Taking a certain amount of genomic DNA as templates, the fusion primer reaction system was configured, and the reaction program was set up for PCR amplification to establish corresponding 72 libraries. The qualified libraries were measured by Illumina sequencing platform in paired-end of 2 × 150 bp reads following the manufacturer’s instructions. The raw data were controlled by removing adaptors using iTools Fqtools fqcheck (v.0.25), cutadapt (v.2.6) and readfq (v1.0), and clean data were collected. Dada2 in qiime2 was used for denoise, resulting in outputs of OTUs and the corresponding characteristic tables. Database silva v138 was used to annotate bacterial species, and qiime2 was applied for alpha and beta diversity analysis. Ordination plots for beta-diversity metrics were generated by principal coordinates analysis (PCoA) based on Bray Curtis, deferentially abundant taxa were identified by the linear discriminant analysis (LDA) effect size (LEfSe) analysis, and graphs visualizations were performed using the OECloud tools. Spearman’ rank was used in correlation analysis, taking thresholds of correlation coefficient *ρ* ≥ 0.40 and *p* ≤ 0.05 and the different folds of average abundance between treatment and control group as ≥ 1.75 or ≤ 0.571.

### 2.6 Quantitative real-time PCR analysis

Intestinal tissue was ground in liquid nitrogen. All samples were performed by real-time PCR using 2 × Talent qPCR PreMix (TIANGEN BIOTECH, Beijing, China). The primers used in this study were designed based on the reference genome of rabbit (mOryCun1.1) (Supplementary Table S3).

### 2.7 Statistical analysis

One-way analysis of variance was used for statistical calculation via SPSS software. The differences among groups were then estimated using the Duncan comparison range tests. The experimental data were showed as the means ± SEM. Value *p* < 0.05 among groups was considered to be statistically significant.

## 3 Results

### 3.1 Effects of ACS on the growth performances of rabbits

**Figure 1:**
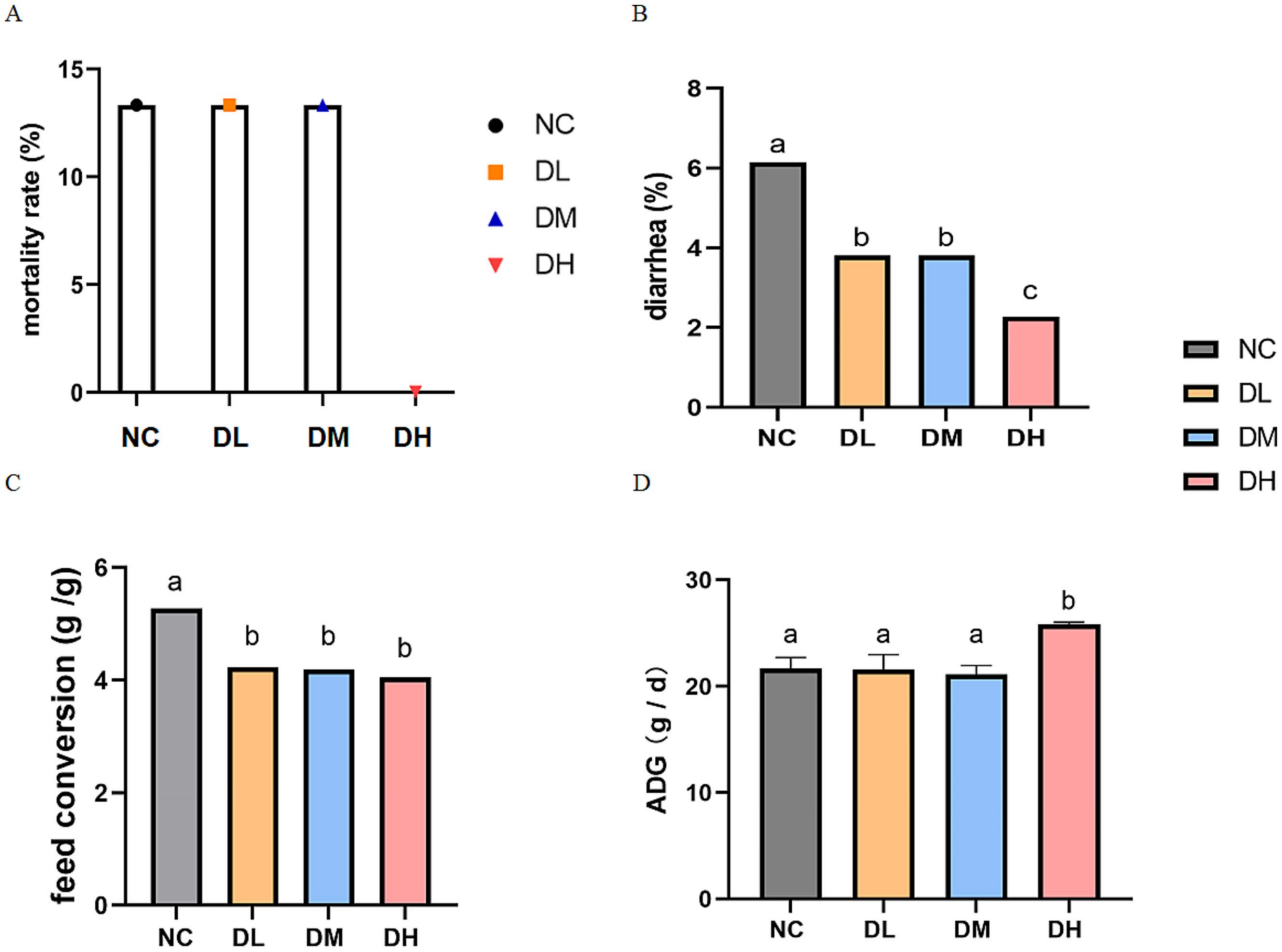
Effects of ACS on rates of mortality (*p* = 0.058 between ACS and NC groups) **(A)** and diarrhea **(B)**, ADG (**C**) and feed conversion ratio **(D)** (*n* = 15) with different letters as significant difference (*p* < 0.05) on the columns.

Compared with control, dietary ACS supplement reduced the diarrhea (*p* < 0.05) and mortality rates of young rabbits, especially mortality rate in the DH group decreased to 0 at the end of experiment (Figures 1A,B). And the ADG in DH groups was increased compared with control group (*p* < 0.05) (Figures 1C,D).

### 3.2 Changes in immunocytes and cytokines after ACS addition

#### 3.2.1 Changes in immunocytes

**Figure 2:**
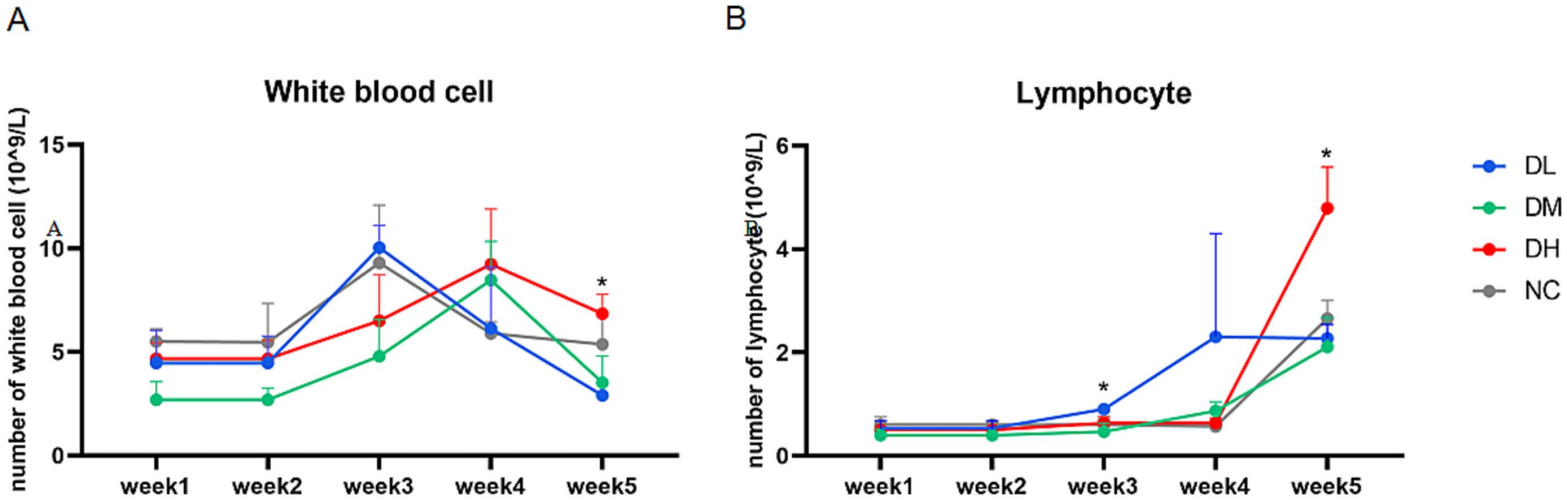
Changes in the numbers of white blood cells **(A)** and lymphocytes **(B)** from the first week to the fifth week (*n* = 3) with star as different significance compared with NC group (*p* < 0.05).

The blood samples of rabbits were collected once a week and examined as soon as possible (Figure 2A). The numbers of lymphocytes (Figure 2B) were significantly higher in DH group compared with NC groups at week 5 (*p* < 0.05). The counts of other blood cells were not significant among groups.

#### 3.2.2 Immune organ indices

**Figure 3:**
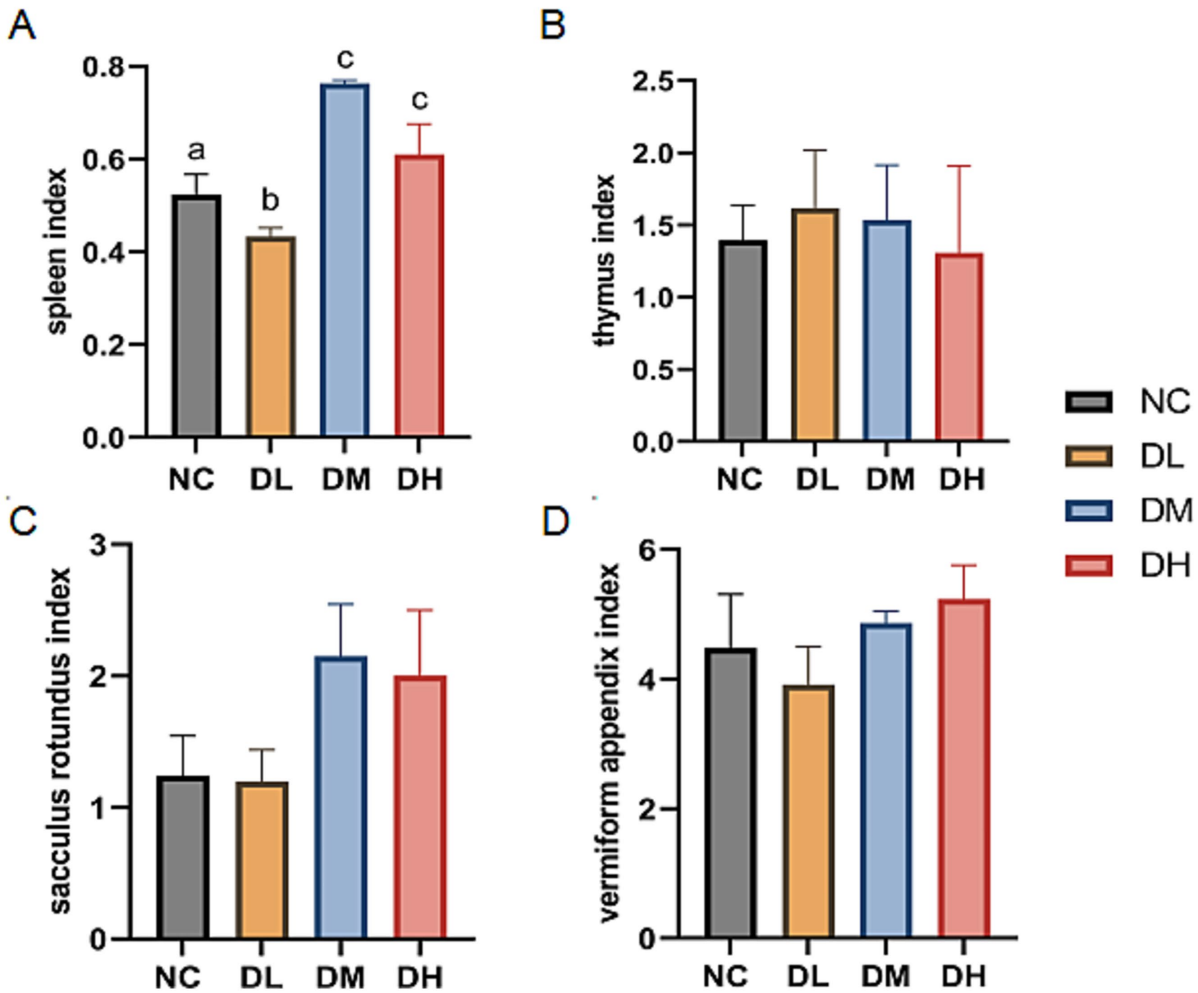
The indexes of spleen **(A)**, thymus **(B)**, sacculus rotumdus **(C)** and vermiform appendix (**D**) in rabbits. The different superscript letters on the columns within the same bar chart were differ significantly (*p* < 0.05). Data were presented as mean ± SEM (*n* = 15).

Immune organs were weighed and those four organ indices were calculated. As shown in Figure 3, the relative weights of the spleen were significantly increased in DM and DH groups after 5 weeks of ACS supplement feeding (*p* < 0.05). However, there was no difference between the groups supplemented with ACS and the controls in thymus, sacculus rotundus, and vermiform appendix indices (*p* > 0.05).

#### 3.2.3 Cytokines in intestine tissues

**Figure 4:**
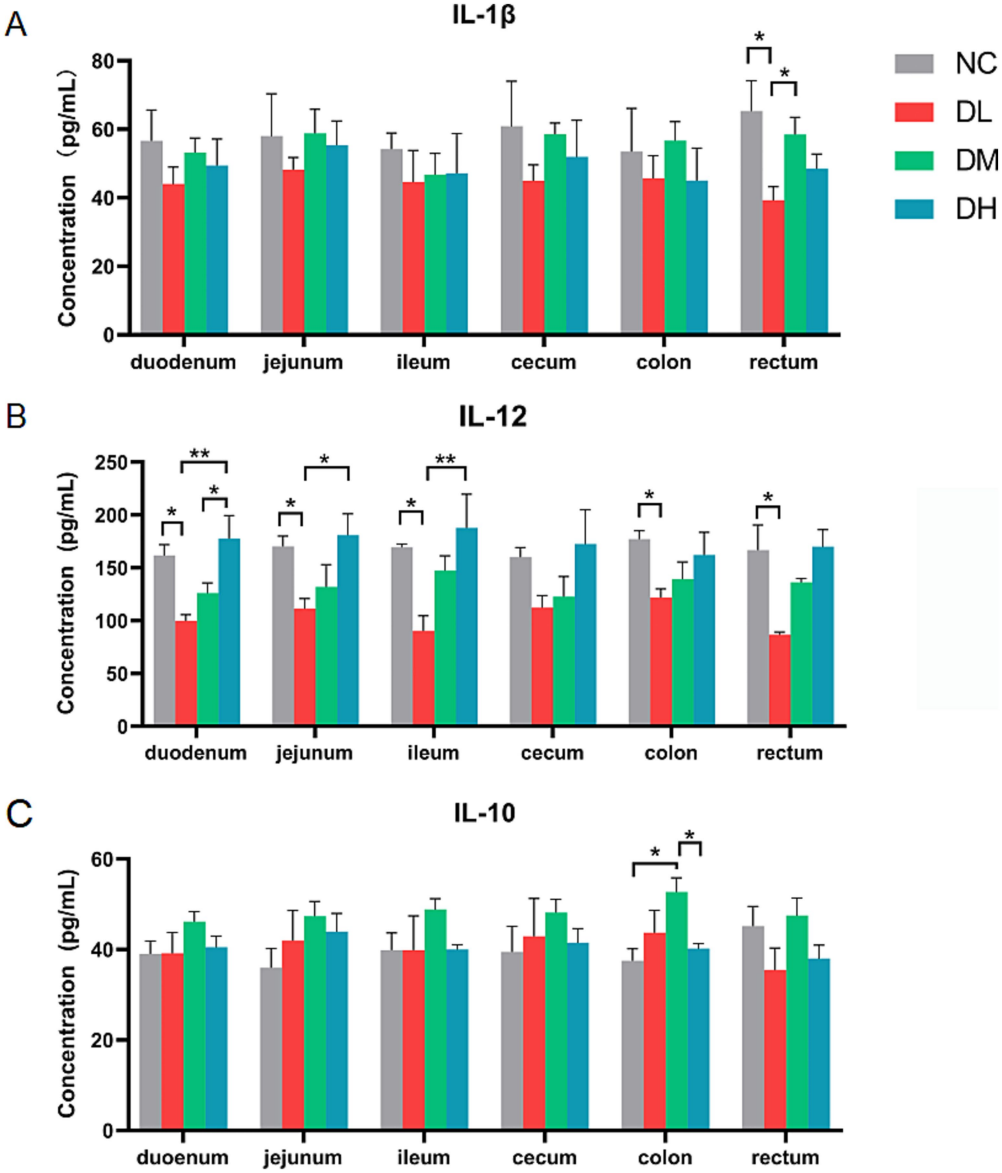
The concentrations of IL-1β **(A)**, IL-12 **(B)**, IL-10 **(C)** in each segment of intestinal tissue. Values were expressed as means ± SE (*n* = 3). Differences were assessed by ANOVA. *p < 0.05. **p < 0.01.

The concentrations of cytokines including IL-1β, IL-10, and IL-12 in six intestinal tissues were determined by ELISA method. The concentration of IL-1β was decreased in DL group compared with control group in rectum (*p* < 0.05) (Figure 4A), and IL-12 was decreased in DL group in five intestines tissues (*p* < 0.05) compared with control group (Figure 4B). Conversely, the contents of anti-inflammatory factor IL-10 in colon was significantly increased in the DM group (*p* < 0.05) (Figure 4C). Taken together, the ACS addition in diet enhanced the immune capacity of young rabbits by increasing the spleen index, the numbers of lymphocytes, and inhibited intestinal inflammation by increasing the concentration of anti-inflammatory factor IL-10 while reducing the concentration of IL-1β and IL-12 in intestinal tissue, suggesting a positive effect of ACS on the immunity of young rabbits.

#### 3.2.4 Quantitative analysis of intestinal barrier and inflammatory associated genes

**Figure 5:**
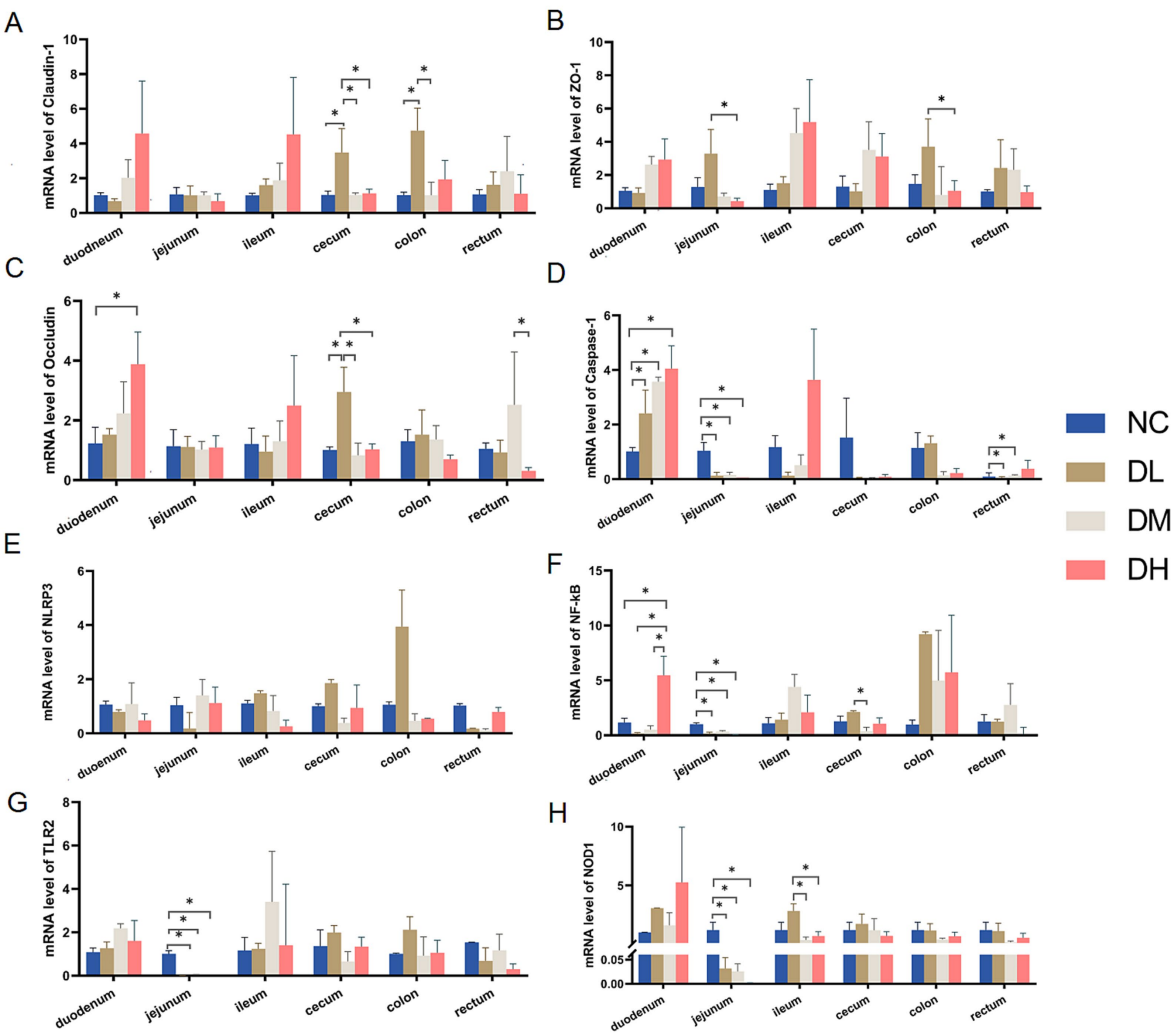
The relative expressions of genes Claudin-1 **(A)**, ZO-1 **(B)**, Occludin **(C)**, Caspase-1 **(D)**, *NLRP3* **(E)**, *NF-κB* **(F)**, *TLR2* **(G)**, *NOD1* **(H)** in each intestinal segment. Values were expressed as mean ± SE (*n* = 3). Differences were assessed by ANOVA. *p < 0.05. **p < 0.01.

Compared with control group, three intestinal barrier genes, Claudin-1, ZO-1 and Occludin, were expressed higher in duodenum, cecum and colon after ACS supplement (Figure 5) (*p* < 0.05). Moreover, ACS addition mainly inhibited the gene expressions of Caspase −1, *NF-κB*, *TLR2*, and *NOD1* in jejunum (*p* < 0.05). In addition, the increased effects on gene expressions of Caspase-1 and *NF-κB* in duodenum could be also observed in ACS groups (*p* < 0.05) (Figures 5D,F).

### 3.3 Effects of ACS on the composition and diversity of gut microbes

#### 3.3.1 Composition of gut microbes

**Figure 6:**
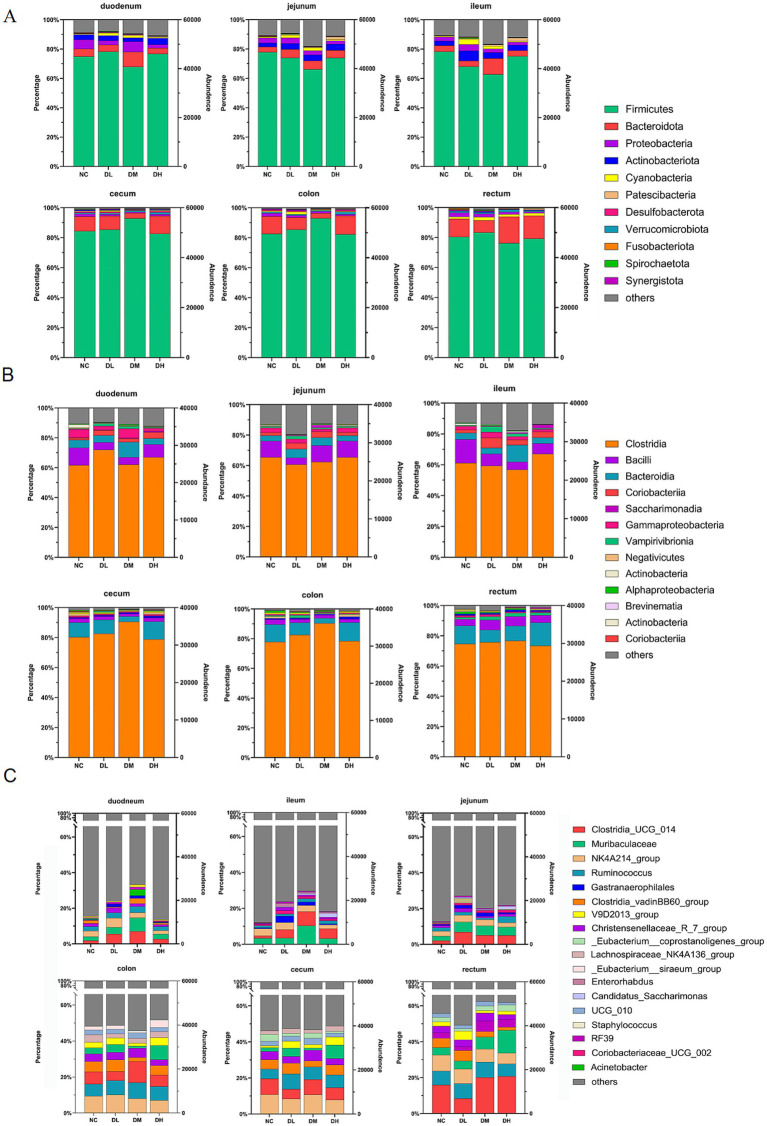
Relative abundances of top 10 bacteria at levels of phyla **(A)**, class **(B)**, and genera **(C)** in six segments of intestinal samples of rabbits supplied with ACS at low (DL), medium (DM) and high (DH) dosages compared with control group (NC) (*n* = 3).

The hypervariable V3–V4 regions of the 16S rRNA gene were sequenced from 72 intestinal libraries with 5,557,460 high quality clean reads via Illumina sequencing platform. It was classified more than 7,000 OTUs, annotations of 28 phylas, 36 class, more than 600 genera. After normalization and filtration, 311 genera were determined with abundance larger than 10 in three samples at least (Supplementary Table S4). Of those, 56 genera occupied 18% of total genera with abundance more than 100. Furthermore, there were 15 genera with abundance higher than 1,000, which occupied 75.33~92.30% of the total abundance in each group. Twelve genera were belonged to phylum Firmicutes. Those high plentiful genera mainly distributed in rectum with abundance percentages more than 50% while the least (less than 17%) was appeared in ileum. The unclassified genus was the richest with abundance up to 23,247 (79.83%) (Supplementary Table S4; Figure 6). The microbial compositions in DM group were much different from NC group. At the phylum level, the abundance of Firmicuts in DM group was increased to 90% compared with NC group in cecum and colon. And it is the same to Bactoidota in DM group in three small intestines (Figure 6A). At the class level, the abundance of Clostridia was higher in DL and DM groups than control group in three large intestines (Figure 6B). At the genus level, the top 18 abundant bacteria genera were shown in Figure 6C, which were increased in DL and DM groups compared with control, Such as *Clostridia_UCG_014* and *Muribaculaceae* were increased in DM group in three small intestines, and *Clostridia_UCG_014* also were elevated in ACS groups from three large intestines.

#### 3.3.2 Effects of ACS on diversity of intestinal flora

**Figure 7:**
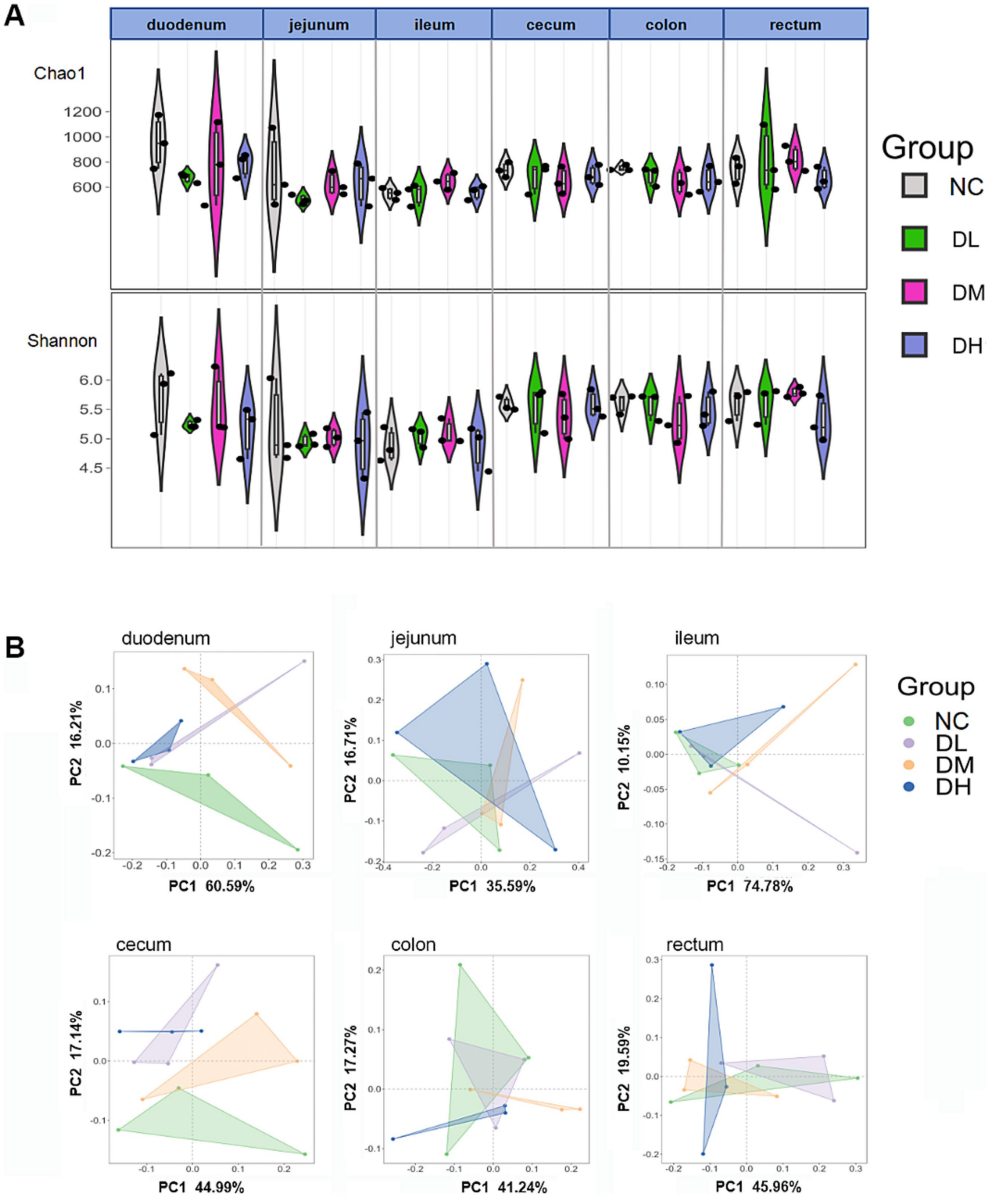
The indices of chao1 and Shannon **(A)** and PCoA **(B)** analysis of six intestine segments (*n* = 3) (*p* > 0.05 among groups in **A,B**, respectively).

The normalized OTU data were employed to analyze bacterial alpha and beta diversity in each group via richness estimators (Chao1) and Shannon diversity indices. There was no significant differences of Chao1 and Shannon index between the ACS groups and the control group (Figure 7A). PC1 and PC2 represent the variance explained ratio of principal coordinates. PC1 values in three small intestines were from 35.59 to 74.78% while PC1 were limited within the range 40–46% in three large intestines (*p* > 0.05) (Figure 7B). These results indicated that ACS could change *β*-diversity of intestinal flora in intestine with ACS addition.

#### 3.3.3 LefSe analysis of microbes in each gut segment

**Figure 8:**
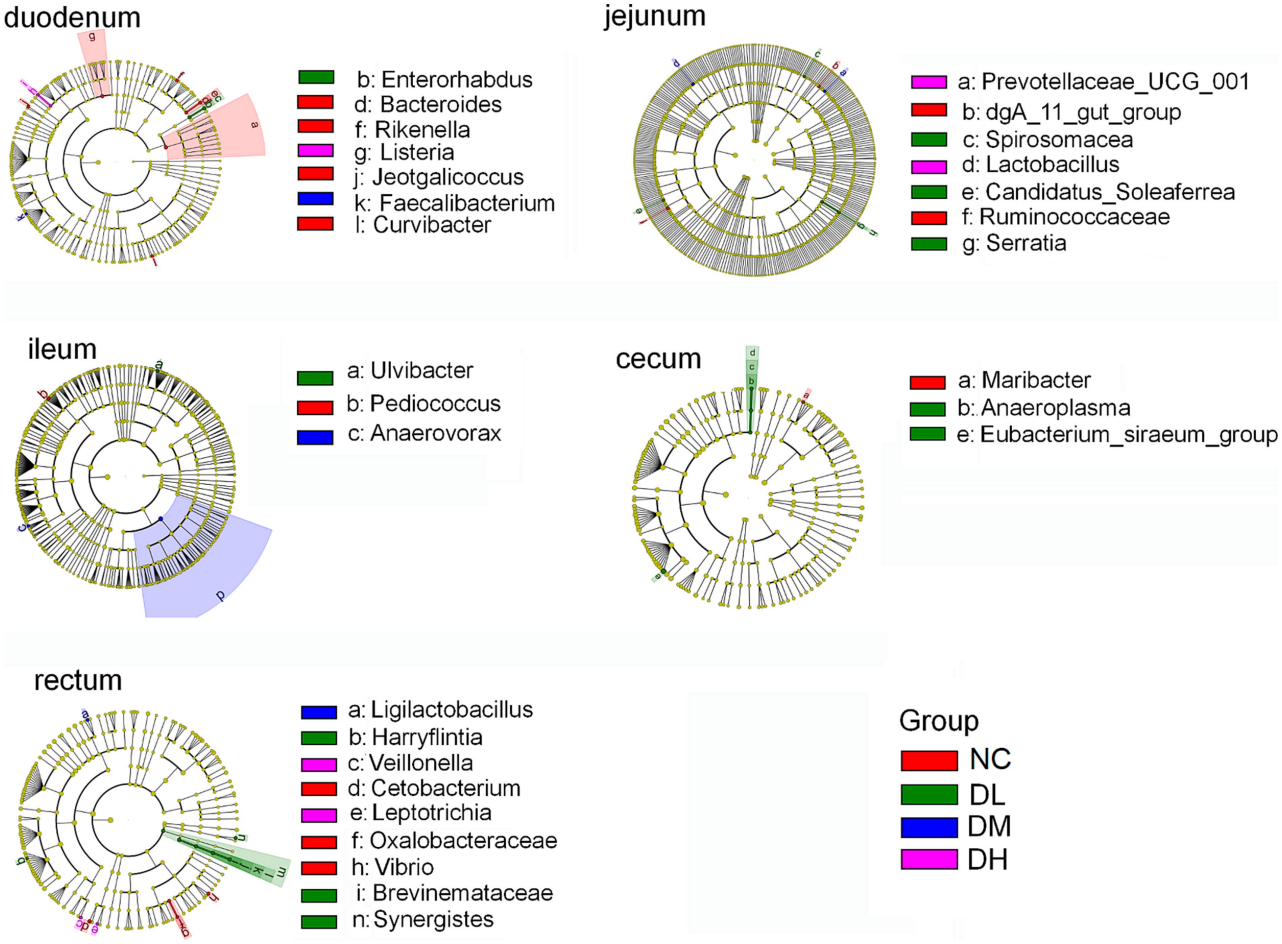
Taxonomic differences of gut flora among different groups. Taxonomic cladogram obtained by LefSe analysis. Bacterial biomarker did not detected form colon digesta (*n* = 3).

To characterize the shifts of microbial composition, we further conducted LEfSe analyses to identify the most prominent taxa based on the relative abundance (*p* < 0.05, logarithmic LDA score ≥ 2). Some biomarkers in intestine were screened (Figure 8), such as *Faecalibacterium* in DM group and *Listeria* in DH group in duodenum. In jejunum, the biomarkers were *Candidatus-soleaferrea*, *Spirosomaceae*, *Serratia* in DL group, *Lactobacillus*, *Prevotellaceae-UCG-001* in DH group. And other biomarkers included *Ulvibacter* in DL group and *Anaerovorax* in DM group in ileum, *Eubacterium-siraeum-group* (LDA > 4), *Anaeroplasma* in DL group in cecum. In rectum, the biomarkers included *Brevinema*, *Harryflintia*, *Synegistes*in DL group, *Ligilactobacillus* in DM group, *Leptotrichia* and *Veillonella* in DH group. Most of these biomarkers were come from Firmicutes and Bacteria phylums, Bacilli and Clostridia classes. These results further supported the dominant lists of Firmicutes and Bacteria in intestine of rabbits.

### 3.4 Functional prediction of intestinal flora

**Figure 9:**
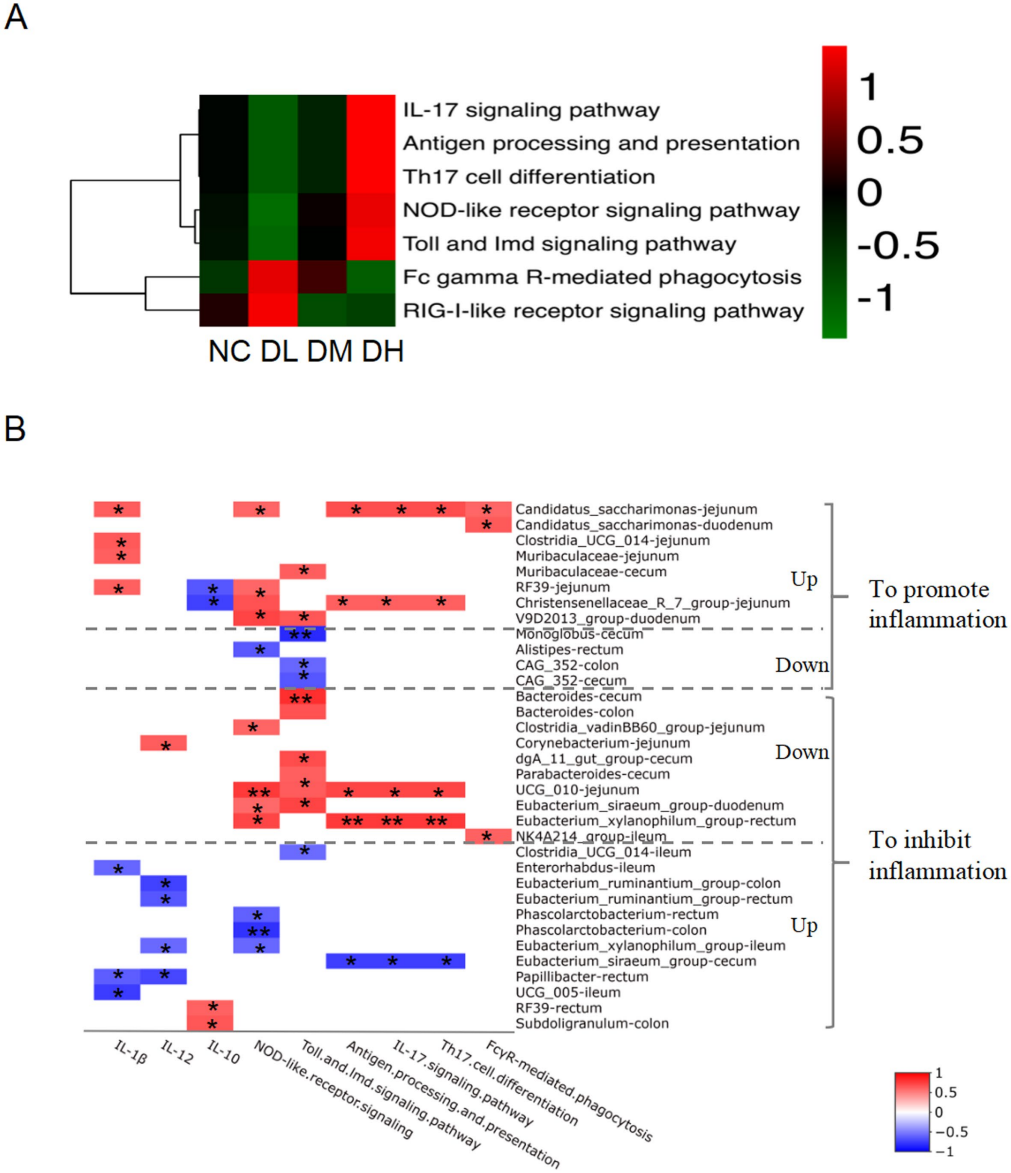
The heat map of KEGG hierarchy pathways enriched in immune system **(A)** of intestinal microbes (*n* = 3) and flora related to immunity **(B)**. The “Up” on the right side of the genera lists denot the increased abundance and “Down” as the decreased abundance of bacteria regulated by ACS treatment. The positive correlation was showed in red color while negative relationship was indicated in blue color (*n* = 3), *p < 0.05. **p < 0.01.

The normalized OTU data were employed to predict the enriched KEGG pathway based on microbial abundance by Picrust2 program. At the first hierarchy of KEGG, the functional prediction of OTU information was concentrated on metabolism with percentage of more than 70%, genetic information processing (10%), cellular process, human disease, environmental information processing and organismal system. We further focused on the enriched immune system (Figure 9A), in which seven pathways, IL-17 signaling, Th17 cell differentiation, NOD-like receptor signaling, Antigen processing and presentation, Toll and lmd signaling were reinforced in DH group compared with NC group, Fc gamma R-mediated phagocytosis and RIG-I-like receptor signaling pathway were gathered in DL group compared with control group. It suggested that the regulation of ACS on the immune activity might mediate via intestinal flora in the gut of young rabbits.

### 3.5 Correlations between bacteria abundance and the changes of cytokines, growth performance, and the predictive abundances of KEGG pathways

To further address the association between the intestinal flora and immune activity, the potential relationship between the intestinal flora abundance and immune pathways that we focused was employed by Spearman’s rank correlation analysis. Total of 30 candidate genera were selected with strong significant relationships. Concentration of three interleukin and the predictive abundance of six KEGG immune pathways were found to be related with those abundances of genera, including IL-17 signaling, antigen processing and presentation, Th17 cell differentiation, NOD-like receptor signaling, Toll and lmd signaling, and FcγR-mediated phagocytosis (Supplementary Table S4). Interestingly, the correlation coefficients were the same among three pathways of Th17 cell differentiation, IL-17 signaling pathway, and antigen processing and presentation. And 24 dominant differential genera between groups were found, which showed clear collaborative effects on immunity responses (Supplementary Table S4; Figure 9B). Among them, nine genera presented promotion effects on intestine inflammation. For example, ACS supplement stimulated genera abundances of *Candidatus_saccharimonas* in jejunum and duodenum, *Clostridia_UCG_014* in jejunum, *Muribaculaceae* in jejunum and cecum, *RF39* in jejunum and *Christensenellaceae_R_7_group* in jejunum, and *V9D2013_group* in duodenum, with positively correlated with the six signaling pathways and concentration of IL-1β, negatively related with concentration of IL-10 (Figure 9B; Supplementary Table S4). Another three genera (*Monoglobus* in cecum, *Alistipes* in rectum, *CAG_352* in colon and cecum) ultimately showed promotion of intestinal inflammation by decreasing abundance of bacteria and negatively related with two pathways of NOD-like receptor and Toll and lmd signaling.

Of those 24 dominant differential genera, 17 genera in ACS groups were represented anti-inflammation effects in intestine. The abundances of nine genera decreased in ACS groups and positively with IL-12 and the six immune signaling pathways, including *Bacteroides* in cecum and colon, *Clostridia_vadinBB60_group* in jejunum, *Corynebacterium* in jejunum, *dgA_11_gut_group* in cecum, *Parabacteroides* in cecum, *UCG_010* in jejunum, *Eubacterium_siraeum_group* in duodenum, *Eubacterium_xylanophilum_group* in cecum, and *NK4A214_group* in ileum. Another 10 genera provided anti-inflammation effects via decreasing richness of abundance and negatively related with the six immune signaling pathways and IL-1β, IL-12, and positively related with IL-10, such as *Clostridia_UCG_014* in ileum, *Enterorhabdus* in ileum, *Eubacterium_ruminantium_group* in colon and rectum, *Phascolarctobacterium* in rectum and colon, *Eubacterium_xylanophilum_group* in ileum, *Eubacterium_siraeum_group* in cecum, *Papillibacter* in rectum, *UCG_005* in ileum, *RF39* in rectum, and *Subdoligranulum* in colon.

## 4 Discussion

The designed ACS decoction improved the growth performances such as the ADG, the decreased rates of death and diarrhea in ACS group, and increased the concentration of IL-10 in high ACS addition while inhibited contents of IL-1β and IL-12 in low ACS addition in intestine tissues. Furthermore, the data from 16S rRNA gene sequencing produced 311 genera from more than 600 genera, due to large amounts of genera presented at low or very low richness. The Firmicutes was predominant phylum in rabbit intestine and many new bacteria were needed to be revealed in future.

**Figure 10:**
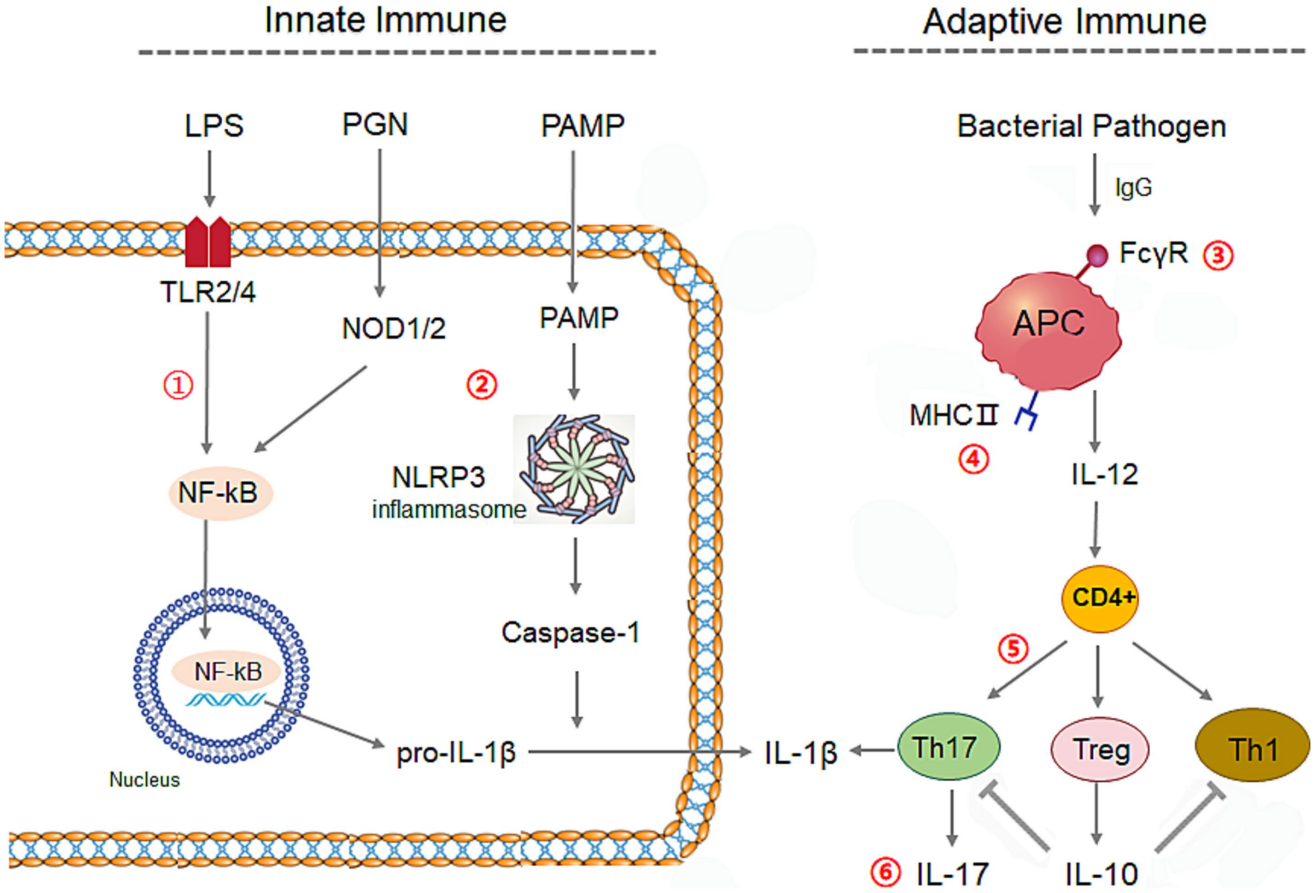
The regulation network by ACS on the innate and adaptive immune systems of young rabbits. The related six immune signaling were listed as numbers ①: Toll and lmd signaling pathway, ②: NOD-like receptor signaling, ③: FcγR-mediated phagocytosis, ④: Antigen processing and presentation, ⑤: Th17 cell differentiation, ⑥: IL-17 signaling pathway, NOD, NOD-like receptor signaling; APC, Antigen-presenting cell; PAMP, pathogen-associated molecular pattern; LPS, Lipopolysaccharide; PGN, peptidoglycan.

We further calculated the correlation coefficients of the relative abundance of each bacterium with the cytokines concentrations, and the predictive abundances in KEGG immune signaling pathways inferred by PICRUSt2 (Douglas et al., 2020), respectively. It obtained 24 differential genera between ACS and control groups, which presented clear collaborative effects on immunity responses on rabbit intestine (Supplementary Table S4; Figure 9B). The related factors and signaling pathways interplay formed complex regulatory networks of innate and adaptive immunity systems in rabbit (Figure 10), in which bacteria in intestine activate NF-κB or NLRP3 inflammasome through Toll and lmd signaling pathway and NOD-like receptor signaling to produce IL-1β in innate immunity, and also stimulate T cells differentiation to produce interleukin by APC via FcγR-mediated phagocytosis and IL-17 signaling pathways in adaptive immunity.

Of those 24 differential genera, nine differential genera between ACS groups and control presented promotion effects on gut inflammation, consisted of *Alistipes*, *CAG*_*352*, *Candidatus*_*saccharimonas*, *Christensenellaceae*_*R*_*7*_*group*, *Clostridia*_*UCG*_*014*, *Monoglobus*, *Muribaculaceae*, *RF39*, *V9D2013*_*group* in digesta of small and large intestines of rabbits (Supplementary Table S4; Figure 10B). Among them, abundances of genus *Alistipes*, *CAG_352* and *Monoglobus* were inhibited by ACS supplement but negatively related with immunity pathways such as NOD-like receptor signaling and Toll and lmd signaling, resulting in promotion on gut inflammation. In ulcerative colitis mice model, the abundance of *Alistipes* is increased and the expressions of NLRP3 and caspase-1 are elevated (Li C. et al., 2024). The inflammation status in rats is related with the enhanced *CAG-352* (Ma et al., 2022). Oral opioids cause the patient fecal richness of *Monoglobus*, and promoting systemic inflammation by activating TLR2/4 (Wang et al., 2022). Another six genura were boosted by ACS and positively related with cytokines concentration and immune signaling pathways. The abundance of *Candidatus saccharimonas* was positively related with IL-1β concentration and five signals including NOD-like receptor signaling, IL-17 signaling, antigen processing and presentation, Th17 cell differentiation, FcγR-mediated phagocytosis pathways. It does not know how the genus affect those signals while it is reported that the abundance of *C. saccharimonas* is increased in the colitis-associated carcinogenesis mice model harbored severe inflammatory in colon (Cruz et al., 2020). Two genera, *Christensenellaceae_R_7_group* and *V9D2013_group,* were also detected to relate with IL-1β and IL-10 and more than one signals in young rabbits. The coprophagy prevention augments cecum inflammation and apoptosis with higher content of IL-1β and abundance of *Christensenellaceae_R-7_group* and *V9D2013_group* in cecum, and many genes enriched in IL-17 signaling, Th17 cell differentiation, NOD-like receptor signaling, antigen processing and presentation, Toll-like receptor signaling pathways based on transcriptome sequencing of cecum from rabbits (Li, et al., 2024), rats and chicken (He et al., 2022; Lin et al., 2023; Ye et al., 2023a). In the present study, the increase of *RF39* in ACS group was positively related with NOD-like receptor signaling together with concentrations of IL-1β and IL10. Broiler chickens with necrotic enteritis show higher mortality and intestinal lesion together with increases of *RF39*, IL-1β, and IL-17/IL-10 ratios in jejunum (Song et al., 2023), and cecum of mice (Zhang et al., 2020b). The elevated richness with 2–3-folds of *Muribaculaceae* and *Clostridia_UCG_014* in ACS groups was significantly related with IL-1β level in young rabbits. It is reported that the abundances of *Muribaculaceae* and *Clostridia UCG 014* are positively correlated with the content of IL-1β in rabbit (Li et al., 2022; Ye et al., 2023a) and in mice (Xie et al., 2024). All of these works indicated that the effects of the nine differential genera had a role to stimulate immunity and reinforce the inflammation response in rabbit intestine.

After ACS supplement, 17 differential genera showed inhibition effects on gut inflammation with strong relationship, such as *Bacteroides* and *Corynebacterium*, etc., in which two genera, *Clostridia_UCG_014* and *RF39* were multifunctional in ileum and rectum. About seven genera were negatively related with interleukin concentration of IL-1β, IL-12, or IL-10 in different gut fragments, including *Corynebacterium*, *Enterorhabdus*, *Eubacterium_ruminantium_group*, *Papillibacter*, *RF39*, *Subdoligranulum*, and *UCG_005*. Taking a protein of *Corynebacterium* as antigen can stimulate IL-12 and Th1 response in immunized sheep and mice (Vale et al., 2016; Barbosa et al., 2024). The abundance of *Enterorhabdus* is negatively associated with colonic IL-1β in mice (Guo et al., 2022). Similarly, phenolic phytonutrients increases *Eubacterium*_*ruminantium*_*group*, reduces inflammation and the concentration of IL-12 in rumen of lambs (Wang et al., 2023). Oral leaf powder from *Moringa oleifera* reduces contents of IL-1β, and increase *Papillibacter* in cecum (Yasoob et al., 2021). *RF39* presents the anti-inflammation effects in chicken and sows (He et al., 2022b; Liu et al., 2022; Chen et al., 2024). N-carbamylglutamate or glutamate increases *Subdoligranulum* and *UCG_005* in colonic digesta, increases IL-10 and IL-1β in piglets (Liu et al., 2023a, 2023b). Furthermore, both of *Subdoligranulum* and *Eubacterium* are butyrate producers. Butyrate is utilized to protect intestinal barrier integrity, restricted pro-inflammatory cytokines to hinder the entry of opportunistic pathogens, and to help a healthy gut environment (Singh et al., 2023).

Those six differential genera were detected to be related with NOD-like receptor signaling or Toll and lmd signaling pathways, consisting of *Bacteroides*, *Clostridia_UCG_014*, *Clostridia_vadinBB60_group*, *dgA_11_gut_group*, *Parabacteroides*, and *Phascolarctobacterium*. Oral supplementation with cranberry polyphenols increases the abundance of *Bacteroides* and TLR2 expression in obese mice (Medina-Larqué et al., 2022). The protection by *Bifidobacterium* is accompanied by an increase of *Clostridia_UCG-014*, reduce the ileum inflammation in mice (Yue Y. et al., 2023). Polysaccharides increases the proliferation of *Clostridia_vadinBB60_group*, the bacteria is contributed to alleviate gut inflammation in mice (Tian et al., 2024). The mulberry 1-deoxynijirimycin increases the abundance of *dgA-11_gut_group*, decreases the inflammation of rabbits (Li S. et al., 2024). The richness of *Parabacteroides* is positively correlated with intestinal integrity while negatively correlated with TLR4 and NF-κB in chicken (Kong et al., 2021). Treatment of electroacupuncture increases fecal richness of *Phascolarctobacterium* and inhibits NLRP3 in rats (Zhou et al., 2022). Other types of three differential genera were related with multiple signalings, including *Eubacterium siraeum group*, *E. xylanophilum group*, and *UCG_010.* The Chinese medicine, Qianshan Huoxue Gao, is reported to improve the inflammation and positively correlated with abundance of *E. xylanophilum group* and *E. siraeum group* (Zhao et al., 2023; Ye et al., 2023b). The family UCG-010 has a role in anti-inflammatory effects after malic acid supplied to sows (Chen et al., 2024).

The decreased *NK4A214_group* in ACS groups was the only genus to be positively related with FcγR-mediated phagocytosis pathway. It is not clear how the bacteria affect FcγR-mediated phagocytosis, but it is detected that the genus is negatively correlate with IL-1β and showing anti-inflammation in Hyplus rabbit cecum (Ye et al., 2023a; Li Z. et al., 2024). In the present paper, the coefficient of *NK4A214_group* abundance with IL-1β concentration was 0.48 although the *p*-value was not significant (*p* = 0.12), which indicated the strain might have a role in the immune regulation of ACS in rabbits. Taken together, all mentioned differential 17 genera bacteria tended to have an anti-inflammation effect in supplement of ACS in intestine of young rabbits.

Numerous researches indicate that the relationship between Chinese herbal medicines and intestinal flora is much complicated. Some components in Chinese herbal medicines affect the structure and abundance of intestinal flora, and improve the intestinal environment for the growth of bacteria. In a similar vein, the vibrant intestinal bacteria can also change the catabolism, absorption, chemical modification, potency of the herbal medicines (Zou et al., 2024). And both of herbal medicines and intestine flora might synergistically regulate the immunity of rabbits. In the aqueous extract from Ciwujia (the monarch herb in ACS decoction), the most potent efficacy of phytochemicals contain polysaccharide, isofraxidin, and eleutheroside E (Lau et al., 2019). Dietary Ciwujia polysaccharide decreases the expressions of *IL-1β*, *IL-6*, and *TNF-α* in spleen of piglets (Han et al., 2014; Fan et al., 2019). The component isofraxidin inhibits IL-1β-induced joint inflammation via the regulation of NF-κB signaling (Lau et al., 2019). And Eleutheroside E suppresses the release of inflammatory cytokine TNF-α and IL-6 in cultured macrophages (Lau et al., 2019). Moreover, Ciwujia reduces the counts of *E. coli*, staphylococci, *Pseudomonads* and *Clostridia* in rabbits (Lauková et al., 2016). And *Astragalus* polysaccharides supplementation significantly decreased the abundance of *Bacteroides* in cecum and closely associated with the alteration of ADG, FCR, TNF-α, IL-1β in chicken and rat (Qiao et al., 2022; Liu Y. et al., 2021; Zhou et al., 2023). Houttuynin, the component of Yuxingcao, harbors activities of antibacterial, anti-inflammatory, antioxidant, antitumor (Cheng et al., 2023; Lau et al., 2019; Liu X. et al., 2021), and regulation of intestinal flora by repressing the TLR4/NF-кB pathway (Zhang et al., 2021; Zhang et al., 2020a). *Indigo Naturalis* (Qingdai in Chinese), has been used for treatment on patients with ulcerative colitis in China, and the major active components are indigo and indirubin (Kakdiya et al., 2024). It can reduce Th17 cell differentiation and IL-17, IL-1β, and TNF-α release in mice with ulcerative colitis (Yue L. et al., 2023). *Glycyrrhizae Radix* et Rhizoma (Gancao in Chinese) is functioned as synergistic effects that enhance the efficacy and reduce the toxicity of other herbs (Li et al., 2019). Huangqi decoction, contained Huangqi and Gancao, increases the diversity and richness in cholestatic mice, alleviated intestinal barrier dysfunction, and decreases the abundance of bacteria such as *Alistipes*, *Bacteroides*, and *Parabacterorides* and hepatic expression of pro-inflammatory factors and NLRP3 (Zou et al., 2021).

In conclusion, dietary supplement with ACS exerted diarrhea and mortality-reducing effects, and improved intestinal immunity by modulating abundance of intestinal flora and immunity responses in young rabbits.

## Data Availability

The data presented in the study are deposited in NCBI SRA repository accession number: PRJNA1214357, https://www.ncbi.nlm.nih.gov/sra/PRJNA1214357.
