# Supplementary material for: Dietary supplement of Acanthopanax senticosus decoction formula improves immune response via intestine flora of rabbits
Source: Front Microbiol. 2025 Mar 4;16:1508280. doi: 10.3389/fmicb.2025.1508280 (PMC11913833; doi:10.3389/fmicb.2025.1508280)
Supplement: Supplementary file 1 [file Table_1.docx]

Table S1. The composition of ACS formula in groups

| Ingredient | Water | Crude protein | Coarse fibre | Crude ash | Calcium % | Total phosphorus | NaCl | Lysine | Cysteine + Methionine |
| --- | --- | --- | --- | --- | --- | --- | --- | --- | --- |
| content | ≤ 14% | ≤14% | ≤7-20% | ≤14% | 0.6-1.5 | ≥ 0.4 | 0.3-0.8% | ≥0.6% | ≥0.5% |

**Table S2. The composition of basal diet of rabbits**

| Dosage of ACS | Ciwujia | Huangqi | Yuxingcao | Qingdai | Gancao | Total |
| --- | --- | --- | --- | --- | --- | --- |
| ACS_DL | 1 g | 1 g | 1 g | 0.3 g | 1g | 4.3 g |
| ACS_DM | 2 g | 2 g | 2 g | 0.4g | 2g | 8.4 g |
| ACS_DH | 3 g | 3 g | 3 g | 0.6 g | 3g | 12.6 g |

**Tbale S3. Primers used in gene expression by RT-qPCR**

| Genes | Primer sequence (5'-3') | Product length |
| --- | --- | --- |
| ZO-1 | F:ACAGCATCCTCCCACCTTTG | 123 bp |
|  | R:GATCACAGTGTGGCAAGCG |  |
| Occludin | F:CACGCTTGCCTGGGACAGAAC | 116 bp |
|  | R:CGTAGCCGTAACCATAGCCATA |  |
| Claudin-1 | GGAGCAAAAGATGCGGATGG | 134 bp |
|  | R：AATTGACAGGGGTCAAAGGGT |  |
| Caspase-1 | F:GCCTGGTCTTGTGATGTGGA | 155 bp |
|  | R:AGCACTCTTGGCTTCGTATT |  |
| NLRP3 | F:CTCTGTGAAGGACTGCTGCA | 280 bp |
|  | TCTCTTCCCGCAGTGTTTCC |  |
| NF-kB | F:CAACCCACCTACCCATCTGT | 123 bp |
|  | R:GCACACGGGGTTTGAAGAAT |  |
| TLR2 | F:GGTTCCCCAGGTTGGATC | 121 bp |
|  | R:GGTCACAAGACAGAGAGGGC |  |
| NOD1 | F :CCTGTCCGATTCTCGTTCCC | 182 bp |
|  | R: CCAGGATTTTGCGAACCTTGT |  |
| GAPDH | F:TGCCACCCACTCCTCTACCTTCG | 163 bp |
|  | R:CCGGTGGTTTGAGGGCTCTTACT |  |
